# Anesthesia-Induced Oxidative Stress: Are There Differences between Intravenous and Inhaled Anesthetics?

**DOI:** 10.1155/2021/8782387

**Published:** 2021-11-27

**Authors:** Thomas Senoner, Corinna Velik-Salchner, Günter Luckner, Helmuth Tauber

**Affiliations:** Dept. of Anesthesia and Critical Care Medicine, Medical University Innsbruck, Innsbruck, Austria

## Abstract

Agents used for the induction of anesthesia have been shown to either promote or mitigate oxidative stress. A fine balance between the presence of reactive oxygen species (ROS) and antioxidants is crucial for the proper normal functioning of the cell. A basal concentration of ROS is essential for the manifestation of cellular functions, whereas disproportionate levels of ROS cause damage to cellular macromolecules such as DNA, lipids, and proteins, eventually leading to necrosis and apoptosis. Increased ROS has been linked with numerous illnesses, such as cardiovascular, immune system, liver, and kidney, and has been shown to promote cancer and accelerate aging. Knowledge of the various pharmacologic agents that increase or reduce oxidative stress may promote a safer way of inducing anesthesia. Furthermore, surgery itself leads to increased ROS production and ischemia/reperfusion injury. Indeed, increased perioperative oxidative stress has been correlated with increased postoperative complications and prolonged recovery. Anesthesiologists care for patients during the whole spectrum of perioperative care and thus are in a unique position to deliver countermeasures to oxidative stress. Using preferentially an induction agent which reduces oxidative stress might lead to better clinical outcomes and fewer postoperative complications. Propofol has been shown in several studies to reduce oxidative stress, which reduces postoperative complications and leads to a faster recovery, and thus might represent the preferred induction agent in the right clinical setting.

## 1. Introduction

Excess production of reactive oxygen species (ROS) has been implicated in the etiology of various chronic diseases such as neurodegenerative diseases, cardiovascular diseases, and cancer [1–4]. ROS comprise both oxygen free radicals, such as superoxide, hydroxyl radicals, and peroxyl radicals, and nonradicals, such as hydrogen peroxide, hypochlorous acid, and ozone [5]. In the majority of cell types, mitochondria are the main source of intracellular oxidant production, while nicotinamide adenine dinucleotide phosphate (NADPH) oxidases (summarized as the NOX family of enzymes) comprise other relevant drivers. Apart from that, numerous other enzymes such as xanthine oxidase, nitric oxide synthase, cyclooxygenases, cytochrome P450 enzymes, and lipoxygenases along with other cell organelles like the peroxisome and endoplasmic reticulum contribute to intracellular ROS production [6]. The main cellular structures afflicted by ROS and reactive nitrogen species (RNS) are DNA, proteins, and lipids. ROS are constantly being generated and constitute a natural part of aerobic life. Indeed, basal levels of ROS are indispensable for the expression of numerous cellular functions, such as defense against invading microorganisms, signal transduction pathways, gene expression, and the promotion of growth or death [7]. Notwithstanding the central relevance of redox reactions, dysregulation of oxidant signaling may initiate or accelerate a host of pathological conditions, resulting in cellular dysfunction. However, the body is endowed with protective measures against ROS via enzymatic (e.g., superoxide dismutase (SOD), catalase (CAT), peroxiredoxin (Prx), and glutathione peroxidase (GSH-Px)) as well as nonenzymatic compounds (e.g., tocopherol/vitamin E, beta-carotene, ascorbate, and glutathione (GSH)) [8].

The last few years have seen a rise in the incidence of surgical procedures being performed each year, which also translates to an increased number of people undergoing general or regional anesthesia [9]. Given this rise in anesthetic procedures being performed, delivering anesthesia in a safe manner becomes of utmost importance. Patients being cared for by anesthesiologists can be exposed to oxidative stress preoperatively, intraoperatively, and postoperatively [10]. Given the fact that the anesthesiologist cares for patients during all three of these phases, he or she has the potential to influence the extent to which the individual patient might be exposed to oxidative stress. However, perioperative oxidative stress is a complex response that comprises also patient and surgical factors. Nonetheless, anesthesiologists are in a unique position to convey countermeasures to oxidative stress and potentially improve the postoperative outcome. As such, a better understanding of the impact anesthetic agents play on oxidative stress and the clinical consequences is urgently needed so as to potentially improve patients' outcomes.

## 2. Oxidative Stress and Postoperative Outcome

The influence of preoperative risk factors and preoperative levels of oxidative stress is imperative in determining the degree of elevation of intraoperative oxidative stress. Additionally, the magnitude of the surgical intervention is a major determinant of intraoperative oxidative stress, with more invasive techniques being associated with increased oxidative stress levels compared with minimally invasive procedures [11]. Furthermore, oxidative stress has been shown to play an important role during different steps of many surgical procedures, such as transplantation (heart, liver, etc.), clamping/unclamping of the aorta (thoracic surgery and abdominal aortic surgery), intermittent inflow occlusion (hepatic and cardiac surgery), and release of a limb tourniquet (orthopedic surgery) [10]. Ischemia-reperfusion injury is a critical condition that often appears in more complex surgeries, with oxidative stress playing a major role in eliciting signaling pathways that lead to the onset of necrosis/apoptosis. During the reoxygenation phase, ROS inflict direct tissue damage and initiate a cascade of deleterious cellular responses which culminate in inflammation and cell death [10].

Preoperative levels of oxidative stress have been shown to predict an increased risk of delayed recovery and complications after surgery [12]. In 186 patients undergoing radical esophagectomy, the preoperative blood d-ROM (derivatives of reactive oxygen metabolite) level, as well as pre- and postoperative plasma FAC, was assessed to determine oxidative stress. Other markers such as white blood cell count, C-reactive protein level, incidence of severe postoperative complications, and postoperative recovery process within 30 days after surgery were also assessed in a double-blind fashion. In patients with elevated preoperative d-ROMs, postoperative levels of white blood cell count and C-reactive protein took longer to return to normal levels. Plasma FAC was decreased postoperatively, and the magnitude was positively correlated with preoperative d-ROM levels. The study further showed that patients receiving propofol had no postoperative decline in FAC, a decreased incidence of severe postoperative complications, and faster uneventful recovery time. An elevated preoperative d-ROM level is associated with increased intraoperative oxidative stress and more postoperative complications with prolonged recovery [12].

Another study analyzed the effect of coronary artery bypass grafting (CABG) on oxidative stress and determined their associations with postoperative complications [13]. Asymmetric dimethylarginine (ADMA), which is associated with an increased risk of coronary artery disease, as well as 8-iso-prostaglandin F_2*α*_ (8-iso-PGF_2*α*_), a marker of increased lipid peroxidation, was measured preoperatively and twice postoperatively: 18-36 h and 5-7 days after surgery. The primary outcomes were in-hospital cardiovascular death and postoperative myocardial infarction.

ADMA increased by 68% at 18–36 h after CABG (*p* = 0.0001) and then decreased by 20% on postoperative days 5–7 (*p* = 0.0001). Similarly, 8-iso-PGF_2*α*_ increased by 30% at 18–36 h post-CABG (*p* = 0.05) and then decreased by 11% on postoperative days 5–7 (*p* < 0.0001). There was a positive correlation between ADMA and 8-iso-PGF_2*α*_ at all time points (*r* = 0.53 at baseline, *r* = 0.81 at 18–36 h, and *r* = 0.80 on postoperative days 5–7, *p* < 0.0001 for all comparisons). Among the 158 patients, 13 (8.2%) patients suffered a postoperative myocardial infarction. These patients had higher ADMA and 8-iso-PGF_2*α*_ levels either at baseline or postoperatively as compared with patients who did not suffer a myocardial infarction. Six patients (3.8%) died during the early postoperative period due to an extensive myocardial infarction. Also, these patients showed higher ADMA and 8-iso-PGF_2*α*_ levels at all three time points compared with patients who survived. The alterations in the measured levels might, at least in part, be explained by enhanced ROS levels after surgery and show that increased oxidative stress is associated with the occurrence of adverse events after cardiac surgery [13].

Since postoperative atrial fibrillation (POAF) is associated with a longer hospital stay, an increased risk of future atrial fibrillation, heart failure, and overall mortality [14], Wu et al. [15] assessed the association of oxidative stress biomarkers (F_2_-isoprostanes, isofurans, and F_3_-isoprostanes) in plasma and urine with incident POAF among 551 cardiac surgery patients. Biomarkers were measured at baseline, the end of surgery, and postoperative day 2. 171 patients developed POAF during follow-up. Oxidative stress biomarkers at baseline were not significantly associated with POAF. By the end of surgery, however, isofurans (both in urine and plasma) and F_3_-isoprostanes (in urine, but not plasma) were significantly associated with an elevated risk of POAF. F_2_-isoprostanes were not associated with a higher POAF risk. At postoperative day 2, plasma isofurans and F_2_-isoprostanes were both associated with an increased risk of POAF. Urine concentrations of these biomarkers as well as F_3_-isoprostane levels in both urine and plasma were not associated with subsequent POAF. There was overall a substantial association, with subject in the top quartile of oxidative stress biomarker having up to 3-fold higher odds of POAF. The results of this study add to the growing evidence supporting the likely key pathogenic role of elevated oxidative stress in POAF [15].

Since evidence of increased oxidative stress and worse postoperative outcome is accumulating, the treating anesthesiologist might ask himself how her or she can reduce the increased oxidative stress that underlies all surgical interventions in order to potentially improve patients' outcomes. Given the many anesthetic agents at the hand of the anesthesiologist, analyzing the most commonly used anesthetic agents regarding their role in generating or mitigating oxidative stress seems reasonable. Thus, we will subsequently analyze first the most common inhaled anesthetics, which will be followed by a thorough analysis of the most commonly used intravenous anesthetics. Only human studies have been analyzed and included in this review.

## 3. Inhaled Anesthetics

Inhaled anesthetics are a chemically and pharmacologically distinct group that includes the potent halogenated ether (isoflurane, sevoflurane, desflurane, and enflurane) and alkane (halothane) volatile anesthetics and the inorganic gaseous anesthetics (nitrous oxide and xenon) [16]. These agents cause a composite pharmacological endpoint which is commonly being described as loss of consciousness, reversible amnesia, and immobility. These diverse effects result from multiple mechanisms which are unique for each individual agent, which vary in their relative potency and efficacy. Indeed, inhaled anesthetics have numerous, agent-specific effects on several molecular targets crucial to neuronal communication and excitability [17, 18]. These various actions work synergistically or individually to bring about the pleiotropic effects innate to inhaled anesthetics [19].

We will subsequently analyze the most common inhaled anesthetics regarding their potential for inducing or mitigating oxidative stress.

**Figure 1:**
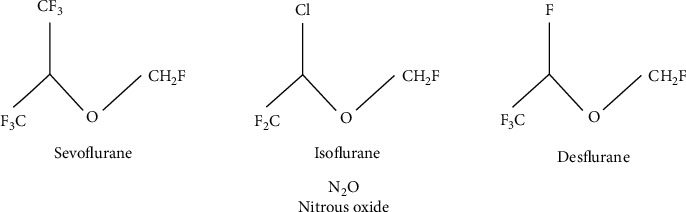
Chemical structures of the inhaled anesthetics discussed in this paper.

Figure 1 represents the chemical structures of the inhaled anesthetics discussed in this paper.

Table 1 summarizes the inhaled anesthetic agents discussed in this paper regarding their effect on oxidative stress.

### 3.1. Sevoflurane

Sevoflurane is a fluorinated methyl isopropyl ether. It is well suited to inhalational inductions because it is minimally pungent and sweet smelling. Because it is completely fluorinated, sevofluranes' blood solubility is very low (among commonly used volatile anesthetics, only desflurane has a lower solubility), and it is about half as potent as isoflurane. Sevoflurane appears to induce the least cerebral vasodilation among the modern agents, and in fact, it has been shown that at 1 MAC (minimum alveolar concentration), cerebral autoregulation and CO_2_ reactivity are preserved, thus making sevoflurane the preferred volatile agent for neurosurgical patients with increased intracranial pressure [20, 21].

The impact of sevoflurane on the cognitive function and the expression of oxidative stress proteins among elderly patients undergoing radical surgery for lung cancer has been prospectively analyzed in a study conducted by Qin et al. [22] and compared with propofol administration. In both groups, serum S100*β* levels and expression of NOX2 and NOX4 proteins were determined in peripheral blood mononuclear cells. 24 hours after surgery, indicators of lung function such as FEV1 (forced expiration in the first second), FVC (forced vital capacity), and VC (vital capacity) were higher in the sevoflurane group as compared to the propofol group (*p* < 0.001, *p* = 0.008, and *p* = 0.002, respectively). At the end of the surgery and 24 hours after surgery, the Mini-Mental State Examination (MMSE) scores were higher in the sevoflurane group as compared to the propofol group (both *p* < 0.001). S100B, a calcium binding protein physiologically produced and released mainly by astrocytes, is considered a sensitive marker of central nervous system damage [23]. Both at the end and 24 hours after surgery, S100B levels were lower in the sevoflurane group (*p* = 0.003 and *p* < 0.001, respectively). Furthermore, levels of markers of oxidative stress (NADPH oxidase subunit NOX2 and NOX4 proteins) in peripheral blood mononuclear cells were also found to be lower in the sevoflurane group (*p* = 0.033, *p* < 0.001, *p* < 0.001, and *p* < 0.001, respectively). The reduction in oxidative stress, as expressed by decreased levels of both NOX2 and NOX4, may mediate the improvement in lung function observed in this study [22].

Another study evaluated the DNA damage effects of sevoflurane and desflurane in human bronchoalveolar cells [24]. Patients undergoing lumbar discectomy surgery were recruited in the prospective study and randomized to either sevoflurane or desflurane for maintenance of anesthesia. Bronchoalveolar lavage samples and peripheral blood samples were collected at baseline (*T*_1_) and at the end of surgery (*T*_2_). Comet analysis was used to examine genotoxic properties. Plasma oxidative DNA damage was measured with 8-hydroxy-2′-deoxyguanosine (8-OHdG), a commonly used biomarker for oxidative stress and carcinogenesis [25]. In both groups, comet parameters at baseline and at the end of surgery were significantly different, in that markedly higher values at *T*_2_ were detected as opposed to baseline levels (*p* < 0.05 for desflurane and *p* < 0.05 for sevoflurane). However, no statistically significant differences have been observed among the 2 groups in *T*_1_ and *T*_2_ comet parameter values (*p* < 0.05). In both the sevoflurane and desflurane groups, plasma 8-OHdG levels were increased at *T*_2_ as compared with baseline levels (*p* < 0.05), while no significant difference emerged between the 2 groups at either time point (*p* < 0.05). In conclusion, both sevoflurane and desflurane may have genotoxic effects on bronchoalveolar cells. Furthermore, the authors observed elevated plasma levels of 8-OHdG, pointing toward increased oxidative damage to DNA [24].

There have been some indications in animal studies that sevoflurane might be neurotoxic in the developing brain, which is partly induced by increased oxidative stress [26–28]. A multicenter, randomized trial sought to investigate whether general anesthesia (GA) in infancy has any effect on the neurodevelopmental outcome compared with regional anesthesia (RA) [29]. In total, 532 infants younger than 60 weeks postmenstrual age who had inguinal herniorrhaphy were randomly assigned to receive either awake-regional anesthesia or sevoflurane-based general anesthesia. The main outcome assessed at 2 years of age was prespecified to be the composite cognitive score of the Bayley-III, which has cognitive, language, and motor scales. The median duration of anesthesia in the GA arm was 54 minutes. For the cognitive composite score, there was equivalence in means between arms (RA-GA: +0.169, 95% CI −2.30 to +2.64). Equivalence was also achieved between arms in the composite motor scores, composite language scores, and composite adaptive behavior scores. In conclusion, this trial demonstrated that a less than one-hour exposure to a sevoflurane GA in infancy does not raise the risk of adverse neurodevelopmental outcome at two years of age [29].

### 3.2. Desflurane

Desflurane is a fluorinated methyl ethyl ether which is identical to isoflurane except for the substitution of a fluorine atom for the *α*-ethyl chlorine. Its complete fluorination results in a markedly reduced blood solubility and potency, while vapor pressure relative to isoflurane is increased to almost atmospheric pressure. The low boiling point of desflurane necessitates a specialized pressurized vaporizer. Desflurane is the most pungent among the inhaled anesthetics and may cause coughing, sialorrhea, breath holding, and laryngospasm. Increased desflurane concentrations may lead to enhanced sympathetic activity with associated tachycardia and hypertension [30]. Because desflurane undergoes almost no metabolism, there is a very low risk of hepatitis and nephrotoxicity.

Some evidence suggests that desflurane may damage the DNA and depress the redox status [31, 32]. A randomized clinical trial sought to investigate whether desflurane is associated with DNA damage and oxidative stress [33]. Blood samples were collected prior to premedication and anesthesia induction (baseline, *T*_1__)_, one and a half hours after anesthesia induction (*T*_2_), and on the morning of the first postoperative day (*T*_3_). The comet assay was used to detect basal and oxidative DNA damage. Biomarkers of lipid peroxidation, such as 4-hydroxynonenal (4-HNE) and 8-iso-prostaglandin F_2*α*_ (8-isoprostane), as well as protein oxidation, were assessed by ELISA. Finally, ferric-reducing antioxidant power (FRAP), an assay which measures the total antioxidant capacity, has been determined to assess the reducing ability of the serum samples. The percentage (%) of DNA strand breaks increased in time (8.2%, 9.5%, and 10.6% at *T*_1_, *T*_2_, and *T*_3_, respectively). The % of oxidized pyrimidines, but not purines, increased in time. Markers of oxidative stress (4-HNE and 8-isoprostane) both increased from *T*_1_ to *T*_3_ [33].

Another small study analyzed the effects of desflurane on oxidative stress by measuring lipid peroxidation (LP), glutathione peroxidase (GSH-Px), erythrocyte superoxide dismutase (SOD), and vitamin E values in blood serum of patients undergoing elective surgery [34]. Blood samples were collected at baseline and 1 and 12 hours following desflurane exposure. LP values significantly differed between baseline and 1 hour postexposure, but not between baseline and 12 hours postexposure. Both GSH-Px and SOD did not differ significantly between the three time points. Vitamin E levels were significantly lower 1 hour postexposure compared to baseline but did not differ between baseline and 12 hours postexposure. These findings suggest that desflurane increases oxidative stress as demonstrated by increased LP values and decreased vitamin E values 1 hour following desflurane exposure [34]. However, the fact that all measured levels returned to baseline values at the 12^th^ hour postexposure casts doubt on the clinical significance of this finding.

The impact of desflurane and sevoflurane on oxidative stress parameters has further been investigated both in mothers and newborns undergoing elective cesarean section [35]. In this randomized study, patients were assigned to either a desflurane (group D) or sevoflurane (group S) group. Blood samples were obtained from mothers at baseline and at the end of surgery, while umbilical artery samples were collected at delivery. Total oxidant status, total antioxidant capacity status, lipid hydroperoxide, and free sulfhydryl levels were measured, and the oxidative stress index was calculated. Preoperative levels did not differ between the two groups. Postoperatively, total oxidant status and oxidative stress index levels were substantially decreased in both groups compared with preoperative levels. Among the two groups, postoperative levels of lipid hydroperoxide, total oxidant status, and oxidative stress index were markedly higher in group D (*p* = 0.003, *p* = 0.005, and *p* = 0.04, respectively) compared with group S. Free sulfhydryl levels and total antioxidant capacity status did not differ significantly among groups. This study demonstrated that both agents induce oxidative stress. However, compared with desflurane, sevoflurane demonstrated more favorable effects in terms of oxidative stress [35].

### 3.3. Isoflurane

Isoflurane was isolated from methyl ethyl ethers in 1965. It shows physical characteristics that are close to the ideal for an anesthetic agent, being highly stable and nonflammable and having a low blood solubility, and it undergoes very little biodegradation. Clinical trials conducted in the 1970's showed that isoflurane leads to a rapid induction and recovery and excellent muscle relaxation and is capable of maintaining blood pressure and cardiac output. It furthermore leads to respiratory depression along with an increase in cerebral blood flow and uterine muscle relaxation [36].

Previous studies have shown that isoflurane may generate the neurotoxicity associated with the pathogenesis of Alzheimer's disease, including accumulation of *β*-amyloid protein and phosphorylation of Tau protein [37–39], and cause neurobehavior deficits [40–42]. A recent study analyzed whether isoflurane causes DNA damage [43]. Since oxidative stress, caspase-activated DNase (CAD), and the p53 signaling pathway are all implicated in DNA damage, ROS, CAD, and p53 were measured in H4 human neuroglioma cells. They were able to demonstrate that isoflurane induces DNA damage by the following mechanisms: (1) isoflurane induced oxidative stress, leading to caspase-3 activation and consequently CAD activation, causing DNA damage, and (2) isoflurane prevented the repair of DNA damage through reduction of p53 levels [43].

A small prospective randomized study compared isoflurane with propofol for maintenance of anesthesia regarding their antioxidant effects on 30 adult patients without comorbidities undergoing elective minor surgery [44]. 15 patients each were randomized to maintenance of anesthesia with either propofol or isoflurane. For the primary outcomes, aqueous plasma oxidizability and total antioxidant performance (TAP) were measured by fluorometry as well as several individual antioxidants by high-performance liquid chromatography. As a secondary outcome, oxidized genetic damage (7,8-dihydro-8-oxoguanine, known as 8-oxo-Gua) was investigated by the comet assay. Maintenance of anesthesia with either propofol or isoflurane resulted in a significant decrease of plasma *α*-tocopherol, but not other antioxidants including uric acid, carotenoids, and retinol. Propofol, but not isoflurane, significantly increased anti-inflammatory/antioxidant plasma *γ*-tocopherol concentration in patients (*p* < 0.001). Both anesthesia types equally enhanced hydrophilic antioxidant capacity and TAP. Furthermore, 8-oxo-Gua remained unchanged during anesthesia in both groups, thus showing that neither of the two anesthetics leads to oxidative DNA damage in this patient group. Thus, the authors concluded that maintenance of anesthesia with either propofol or isoflurane increases both hydrophilic and total antioxidant capacity in plasma, but only propofol anesthesia increases plasma *γ*-tocopherol concentration [44].

### 3.4. Nitrous Oxide

Nitrous oxide (N_2_O) was first used in the late 18^th^ century and has gained wide popularity in the anesthetic world because of its fast uptake and elimination, in addition to its analgesic effects [45]. N_2_O is the least potent among the currently available inhalation agents. It seems to act on multiple targets, including noncompetitive inhibition of N-Methyl-D-Aspartate (NMDA) and non-NMDA glutamate receptors, nicotinic acetylcholine (nACH) receptors, and gamma-aminobutyric acid (GABA) receptors, along with activity on calcium and potassium channels [46, 47]. The analgesic effects of N_2_O is caused by the inhibition of supraspinal NMDA receptors [48]. The low toxicity of modern anesthetic agents and increasing evidence of the deleterious effects of N_2_O have led to a steady decline in the use of this agent in general anesthesia [49, 50]. Various types of cytogenetic damage, including increased sister chromatid exchange [51], chromosomal aberrations [52], and DNA single-strand breaks [53], have been observed in operating room staff exposed to N_2_O. A study compared nurses who had been exposed to N_2_O to a group of volunteer nurses not being exposed to the agent [54]. The numbers of binucleated cells carrying between one to four micronuclei were recorded individually, and the means of the total cytokinesis-block micronucleus assay (CBMNt) and total ARA-enhanced micronucleus assay (CBMNAt) frequencies were compared between the exposed and control populations. The CBMN assay measures DNA damage, cytostasis, and cytotoxicity in different tissue types, including lymphocytes [55]. The mean frequency of CBMNt in lymphocytes of nurses with N_2_O exposure was significantly increased over the controls. A linear correlation between duration of exposure and the CBMNt or CBMNAt frequencies showed that cytogenetic damage might be associated with chronic ambient N_2_O exposure. In conclusion, this study showed that chronic N_2_O exposure may lead to some trans-acting DNA damage or increased error-prone DNA repair in *in vitro* cultivated lymphocytes. These lesions would appear *in vitro* as micronuclei or some other cytogenetic damage [54].

## 4. Intravenous Anesthetics

Intravenous anesthesia can be traced back to the mid-17^th^ century, where it has been used both in animals and humans and entered the era of modern anesthesia with the release of thiopental in 1936 [56]. Since then, the pharmacodynamics and pharmacokinetics of intravenous anesthetics have been studied in much greater detail. Building on this knowledge and the availability of short-acting drugs has shifted the attention of the anesthesiologist to the administration of anesthesia not based on the needs of the population but on the individual needs of the patient.

We will subsequently analyze the most common intravenous anesthetics regarding their potential for inducing or mitigating oxidative stress.

**Figure 2:**
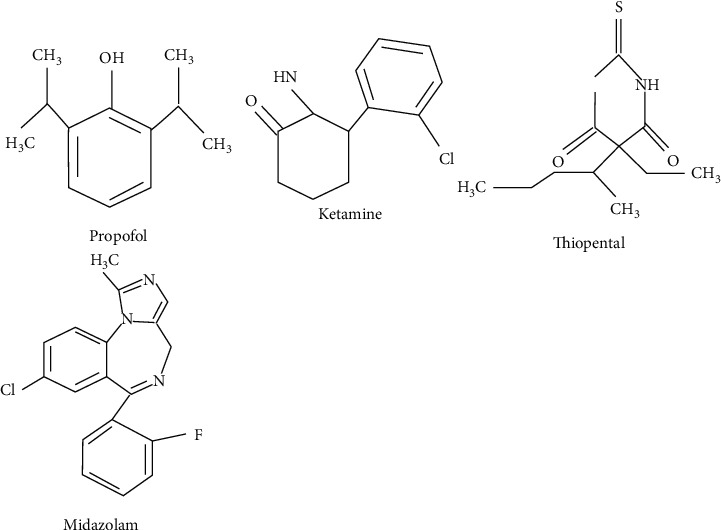
Chemical structures of the intravenous anesthetics discussed in this paper.

Figure 2 represents the chemical structures of the intravenous anesthetics discussed in this paper.

Table 2 summarizes the intravenous anesthetic agents discussed in this paper regarding their effect on oxidative stress.

### 4.1. Propofol

Propofol belongs to a group of alkylphenols which have all in common that they are particularly lipid soluble and insoluble in an aqueous solution. It acts mainly by enhancing GABA-induced chloride current through its binding to the *β*-subunit of GABA_A_ receptor [57, 58]. Furthermore, acetylcholine release in the hippocampus and prefrontal cortex is diminished. The *α*_2_-adrenoreceptor system also appears to be involved in mediating the sedative effects of propofol [59].

Propofol has been shown to possess antioxidant properties, and a reduction of oxidative stress has been associated with an improved postoperative outcome, thus making it an ideal anesthetic in clinical practice [10, 60, 61]. Indeed, a study took it upon itself to analyze whether propofol exerts neuroprotective effects [62]. Human SH-SY5Y cells were pretreated with ferric ammonium citrate (FAC) and then treated with propofol. FAC pretreatment induced cell damage. When these cells were treated with propofol (5 *μ*M) for 6 hours, increased cell viability could be observed. To assess whether this effect correlated with iron levels, the cellular iron levels were additionally tested. Propofol decreased the cellular iron concentration dramatically, indicating that propofol suppresses the iron increase induced by FAC. Additionally, the authors assessed the ROS and malondialdehyde (MDA) levels of SHSY5Y cells. The results showed that FAC significantly increased ROS and MDA levels. Propofol significantly decreased ROS levels but did not significantly decrease MDA levels. Further, the authors noted that the FAC-induced increase in ROS was closely associated with a decrease in the mitochondrial membrane potential (MMP), which constitutes an essential component in the process of energy storage during oxidative phosphorylation [63]. Propofol was able to suppress the decrease of MMP induced by FAC. FAC induced increased cellular apoptosis, which could be significantly inhibited by treatment with propofol. Treatment with FAC leads to an increase in the expression of inflammatory markers, such as interleukin- (IL-) 6 and cyclooxygenase 2. Propofol significantly inhibited the expression of both proteins, indicating that propofol might influence the inflammatory pathway. In summary, this study demonstrated that propofol influences both levels of oxidative stress and inflammation [62]. The antioxidative properties of propofol were also observed in human cardiac cells, and the protective effect was attributed to an activation of the JNK signaling pathway [64].

A prospective, randomized trial by Guo et al. [65] evaluated in 60 patients undergoing aneurysm clipping the role of propofol postconditioning on oxidative stress and postoperative cognitive function. The patients were randomized into a propofol postconditioning group or a sevoflurane group. In both groups, sevoflurane (0.5%-2%) was used for maintenance of anesthesia. In the propofol postconditioning group, the concentration of sevoflurane was diminished following temporary clip removal, and propofol was subsequently started. Blood samples were taken at 6 time points: before induction, immediately after clip removal, at the end of the operation, 24 hours postsurgery, and 3 and 7 days postsurgery. Oxidative stress (hydroxyl radicals (^·^OH), 8-isoprostane, *α*-tocopherol, *γ*-tocopherol, and SOD) and cognitive function were measured. Between the end of the operation to 7 days postsurgery, both ^·^OH and 8-isoprostane levels were markedly lower in the propofol group, while levels of *γ*-tocopherol and SOD, markers of antioxidant capacity, increased significantly in the propofol group. To assess DNA damage, a cytokinesis-block micronucleus test was performed. Postoperatively, micronuclei as well as nucleoplasmic bridges were seen more frequently in the sevoflurane group. Finally, the propofol postconditioning group had markedly higher scores on cognitive function tests compared with the sevoflurane group after the surgery [65].

### 4.2. Ketamine

Ketamine, an arylcyclohexylamine related to phencyclidine, was developed in the 1960's and was originally produced as a racemic mixture of the R and S enantiomers. In some European countries, a single enantiomer formulation (S (+)) is available which is more potent in most clinical and experimental settings. Ketamine has been described to cause a sense of disconnection from the environment, leading to the term “dissociative anesthesia” [66]. The key site(s) of ketamine action have not been entirely elucidated; however, ketamine has been identified as an antagonist at the NMDA receptor in the brain and spinal cord [67]. The role of NMDA receptors in mediating analgesia is supported by experimental data, while their role in inducing anesthesia is unclear [68]. At clinically relevant concentrations, ketamine also inhibits nAch receptors, which could contribute to analgesia but do not seem to affect its sedative properties [69]. Furthermore, ketamine influences also Na^+^ channels and binds to *μ*- and *κ*-opioid receptors, thus possessing some local anesthetic properties [70]. However, the minimal effect of naloxone on ketamine actions suggests a limited role for these receptors. Ketamine has unique properties combining depressed consciousness with an increased sympathetic tone, thus making it a useful induction agent in patients with hypovolemia [71].

The antioxidant properties of ketamine have been studied in a head-to-head comparison with propofol in 80 patients undergoing general surgeries [72]. 40 patients each were randomized either in the ketamine group, where general anesthesia was induced by intravenous injection of ketamine, and a propofol group, where propofol constituted the induction agent. Blood samples were obtained 15 minutes after induction, and the following oxidative markers were assessed: serum SOD activity, GSH-Px, CAT and levels of LP, total antioxidant capacity (TAC), and total thiol molecules (TTM). This study demonstrated that propofol significantly increased TTM and TAC (*p* = 0.001 and *p* = 0.003, respectively) and decreased LP (*p* = 0.014), SOD (*p* = 0.047), and GSH-Px (*p* = 0.037) activity compared to the ketamine group. The level of CAT did not differ significantly between the two groups. Thus, propofol possesses greater antioxidant capacities compared with ketamine [72].

### 4.3. Thiopental

Barbiturates were discovered in the early 20^th^ century, with hexobarbital being the first agent of this class. In 1934, thiopental became preferred clinically due to its rapid onset of action and short duration. Apart from the action on the GABA_A_ receptor, the mechanism of action of barbiturates is largely unknown. NMDA receptors might also play a role in the effects of barbiturates [73]. Barbiturates enhance the synaptic actions of inhibitory neurotransmitters and block the synaptic actions of excitatory neurotransmitters in the central nervous system (CNS) [74]. GABA is the main inhibitory neurotransmitter in the CNS, and the GABA_A_ receptor is the only site proven to be involved in barbiturate-induced anesthesia [75]. The inhibition of synaptic actions of excitatory neurotransmitters involves mainly glutamate and acetylcholine. Thiopental may exert GABA-independent effects on the glutaminergic-NMDA system. Two studies demonstrated that thiopental decreases extracellular glutamate levels in the CNS and decreases NMDA-gated currents in a concentration-dependent manner [73, 76].

To date, no studies exist which analyzed the antioxidant effects of thiopental on humans. However, animal and *in vitro* studies exist which suggest that thiopental possesses antioxidant capacities, even though the effect has been observed to be smaller than that of propofol [77].

### 4.4. Midazolam

The benzodiazepines are a class of drugs often used in anesthesia as anxiolytics, sedatives, and hypnotics. In clinical practice, midazolam is frequently used immediately before induction of anesthesia. Like most intravenous anesthetics, benzodiazepines exert their action through GABA_A_ receptors [78]. They are widely prescribed, and addiction to these drugs is a worldwide concern. The *α*_2_- and *α*_3_-subunits of the GABA_A_ receptors are implicated as key mediators of the reward-related effects of benzodiazepines [79]. All benzodiazepines have hypnotic, sedative, anxiolytic, amnesic, anticonvulsant, and centrally produced muscle-relaxing properties. The mechanism of action of benzodiazepines is reasonably well understood [80]. The *α*-subunit of the GABA receptor occurs in six isoforms (*α*_1_-*α*_6_) [81]. Sedation, anterograde amnesia, and anticonvulsant properties are mediated via *α*_1_-subunits [80], and anxiolysis and muscle relaxation are mediated via the *α*_2_-subunits.

The effects of midazolam on inflammation and oxidative stress have been compared with propofol in children with congenital heart disease undergoing cardiac surgery [82]. 32 children were randomly assigned to a midazolam or propofol (both combined with low dose fentanyl) group. Blood samples were collected preoperatively (*T*_0_), at 2 h following the release of the aorta cross-clamp (*T*_1_), and at 24 h after operation (*T*_2_), and the following oxidative stress markers are measured: IL-6, IL-8, SOD, and MDA levels. After cardiopulmonary bypass, IL-6 and IL-8 levels were markedly increased in both groups at *T*_1_ and *T*_2_ compared with *T*_0_. However, the propofol group exhibited lower IL-6 and IL-8 levels at *T*_1_ and *T*_2_ compared with the midazolam group. Similarly, MDA levels were significantly increased, while SOD levels were significantly decreased at *T*_1_ and *T*_2_ in both groups. The propofol group exhibited lower MDA levels and higher SOD levels compared with the midazolam group. Thus, this study showed that propofol exerts greater potential to decrease inflammation and oxidative stress compared with midazolam.

## 5. Conclusions

Surgical interventions have been associated with increased levels of oxidative stress, and the increase has been closely correlated with the extent of the surgical procedure. Furthermore, increased levels of oxidative stress have been associated with worse clinical outcomes and increased postoperative complications. Given the magnitude of anesthesia being delivered each day, procedural safety and few postoperative complications should be a top priority for every anesthesiologist. The anesthesiologist's armamentarium has broadened significantly over the past decades, and several induction methods using different anesthetic agents are at the disposal of the anesthesiologist. However, as highlighted in this review, some anesthetic agents have a greater potential to induce oxidative stress as compared with others, with some agents even exerting antioxidative effects. Among the most commonly used induction agents, propofol seems to possess the greatest benefits in terms of reducing oxidative stress. Since propofol constitutes the most widely used induction agent in the developed world, the antioxidative properties it possesses certainly come in handy.

Given the impact that anesthesiologists can have on the patients' well-being, we should definitely care about anesthesia-induced oxidative stress.

## Figures and Tables

**Table 1:** Inhaled anesthetic agents investigated regarding their effect on oxidative stress.

Study reference	Anesthetic agent	Studied population	Main effect on oxidative stress
[22]	Sevoflurane	Human study	↓ S100B levels, NOX2 and NOX4 protein levels compared with propofol treatment
[24]	Sevoflurane, desflurane	Human study	↑ Genotoxic effects on bronchoalveolar cells ↑ Plasma levels of 8-OHdG
[29]	Sevoflurane	Human study	Not ↑ risk of adverse neurodevelopmental outcome in infants
[33]	Desflurane	Human study	↑ % of DNA strand breaks, ↑ % of oxidized pyrimidines, ↑ levels of 4-HNE and 8-isoprostane, ↓ % of oxidized pyrimidines
[34]	Desflurane	Human study	↑ LP values and ↓ vitamin E levels 1 hour following desflurane exposure
[35]	Sevoflurane, desflurane	Human study	Significantly greater ↑ in postoperative LOOH, TOS, and OSI levels in desflurane-treated pts compared with sevoflurane
[43]	Isoflurane	Human study	↑ DNA damage, inhibits repair of DNA damage by reducing p53 levels
[51]–[54]	Nitrous oxide	Human study	↑ Sister chromatid exchange, chromosomal aberrations, DNA single-strand breaks

4-HNE: 4-hydroxynonenal; 8-OHdG: 8-hydroxy-2′-deoxyguanosine; DNA: deoxyribonucleic acid; LOOH: lipid hydroperoxide; LP: lipid peroxidation; NOX: NADPH oxidase; OSI: oxidative stress index; PTS: patients; TOS: total oxidant status.

**Table 2:** Intravenous anesthetic agents investigated regarding their effect on oxidative stress.

Study reference	Anesthetic agent	Studied population	Main effect on oxidative stress
[62]	Propofol	Human study	↑ Cell viability, MMP, ↓ ROS, IL-6, and COX2 levels
[64]	Propofol	Human study	Activation of the JNK signaling pathway
[65]	Propofol, sevoflurane	Human study	↓ ^·^OH, 8-isoprostane, micronuclei, nucleoplasmic bridges, ↑ *γ*-tocopherol, SOD compared with sevoflurane
[72]	Propofol, ketamine	Human study	↓ LP, SOD, GSH-Px, ↑ TTM, TAC compared with ketamine
[82]	Propofol, midazolam	Human study	↓ IL-6, IL-8, MDA and ↑ SOD levels compared with midazolam

COX: cyclooxygenase; GSH-Px: glutathione peroxidase; IL: interleukin; JNK: c-Jun N-terminal kinase; LP: lipid peroxidation; MDA: malondialdehyde; MMP: mitochondrial membrane potential; ^·^OH: hydroxyl radical; ROS: reactive oxygen species; SOD: superoxide dismutase; TAC: total antioxidant capacity; TTM: total thiol molecules.

## Abbreviations

8-OHdG: 8-Hydroxy-2′-deoxyguanosine
ADMA: Asymmetric dimethylarginine
CABG: Coronary artery bypass grafting
CNS: Central nervous system
d-ROMs: Derivatives of reactive oxygen metabolites
GABA: Gamma-aminobutyric acid
LP: Lipid peroxidation
MDA: Malondialdehyde
MMP: Mitochondrial membrane potential
N_2_O: Nitrous oxide
nACH: Nicotinic acetylcholine
NADPH: Nicotinamide adenine dinucleotide phosphate
NMDA: N-Methyl-D-Aspartate
NOX2: Nicotinamide adenine dinucleotide phosphate oxidase isoform 2
ROS: Reactive oxygen species
RNS: Reactive nitrogen species
SOD: Superoxide dismutase.
